# Long-Term Suicide Risk of Children and Adolescents With Attention Deficit and Hyperactivity Disorder—A Systematic Review

**DOI:** 10.3389/fpsyt.2020.557909

**Published:** 2020-12-21

**Authors:** Peter Garas, Judit Balazs

**Affiliations:** ^1^Mental Health Sciences School of Ph.D., Semmelweis University, Budapest, Hungary; ^2^Institute of Psychology, Eötvös Loránd University, Budapest, Hungary; ^3^Department of Psychology, Bjørknes University College, Oslo, Norway

**Keywords:** ADHD, suicidality, follow-up, review, children, adolescent

## Abstract

**Background:** Attention deficit and hyperactivity disorder (ADHD) is one of the most common mental disorders in childhood. Recently, several studies showed the high suicide risk of patients with ADHD; however, most of these studies had a cross-sectional design.

**Aims:** The aim of the current research is to complete a systematic review of published studies which investigate the suicide risk of ADHD patients with longitudinal design.

**Methods:** The systematic search was made on OVID Medline, PsychInfo, PubMed, Scopus, and Web of Science. The search terms were (ADHD OR attention deficit hyperactivity disorder) AND (suicide OR suicidal OR suicidality) AND (follow-up OR longitudinal study OR prospective study). The inclusion criteria were as follows: written in English; the participants were under 18 years at baseline; longitudinal, prospective studies; ADHD population at baseline and at follow-up; and suicide behavior as a primary outcome. The exclusion criteria were as follows: the study did not contain empirical data and reviews/meta-analyses and studies which aimed to investigate the drug treatment efficacy of ADHD.

**Results:** After the screening process, 18 papers were included in the systematic review. Ten articles were altogether published in the last 5 years. The range of follow-up periods varied between 2 and 17 years. Several different assessment tools were used to investigate the symptoms and/or the diagnosis of ADHD and the suicidal risk. Nine studies enrolled children aged under 12 at baseline, and three studies used birth cohort data, where there was no strict age-based inclusion criteria. A total of 17 studies found a positive association between ADHD diagnosis at baseline and the presence of suicidal behavior and/or attempts at the follow-up visits.

**Limitations:** The main limitation of this review is the methodological heterogeneity of the selected studies. A further limitation is the relatively low number of studies that examined a population with balanced gender ratios. Additionally, only one study published data about the treatment of ADHD. Finally, though we carefully chose the keywords, we still may be missing some relevant papers on this topic.

**Conclusions:** In spite of the methodological diversity of the included studies, the results of the current systematic review highlight the importance of screening suicidality in the long term in patients with ADHD. Therefore, further studies that compare the suicidal risk of treated and untreated groups of ADHD patients in the long term are needed.

## Introduction

Attention deficit and hyperactivity disorder (ADHD) is one of the most common child and adolescent psychiatric disorders, with a prevalence rate of 3.4% (CI 95% 2.6–4.5) (1). The core symptoms are inattention, impulsivity, and hyperactivity, based on DSM-5 (2). There is growing evidence that ADHD has a significant negative effect on quality of life (QoL) as predicted by the symptom level and impairment. In connection with the QoL aspect, ADHD is associated with social–relationship problems (3), educational problems (4), and increased risk of substance abuse (5) and criminality (6). All these can reduce the effective treatment of ADHD (7). Several studies have suggested that long-term methylphenidate treatment in ADHD could reduce depression and suicidality (8). Numerous research studies have highlighted that comorbid conditions have an important role in the lower QoL of patients with ADHD (9). Children with ADHD had a significantly higher rate of comorbidities than individuals without ADHD (10). Among the most common comorbidities in ADHD are major depression, conduct disorder, anxiety disorder, and substance use disorder (11).

Suicide deaths are the second most common cause of death among females and third among males in the ages 15–29 years worldwide (12). Recently, several studies investigated the correlation between ADHD and suicidality, and even more reviews and meta-analyses were published with the inclusion of these studies (13–18). These reviews concluded a positive association between ADHD and suicidal behavior in all age groups compared; however, the relationship between suicide thoughts and behavior and ADHD is still unclear, i.e., the mediator and moderator factors, mainly the role of comorbidities.

All these previous reviews and meta-analyses focusing on the topic of ADHD and suicidality mainly included studies with a cross-sectional design. A very recent systematic review and meta-analysis by Septier et al. (17) collected cross-sectional and longitudinal data among child, adolescent, and adult studies. They focused only on the cross-sectional relationship, and baseline data were extracted from the longitudinal studies. The results found a significant association between ADHD and suicidal attempts, suicidal ideations, suicidal plans, and completed suicide.

The main aim of the current study was to identify all previously published studies which investigated the connection between ADHD and suicide thought and behavior with a longitudinal design, where the baseline data were available from age under 18 and that assessed ADHD participants. Furthermore, our aim was to provide a systematic overview about these identified studies according to findings, methods, and design.

## Methods

The literature search was made on the 24th of March 2019 on the following scientific electronic databases: OVID Medline, OVID PsychInfo, PubMed, Scopus, and Web of Science. The following search terms were calculated using a Boolean operator (to combine the categories, we used AND terms; to aggregate the subcategories we used OR terms): (ADHD OR attention deficit hyperactivity disorder) AND (suicide OR suicidal OR suicidality) AND (follow-up OR longitudinal study OR prospective study). To organize the articles and to manage the filtration, we used EndNote X8 software. The inclusion criteria of the systematic search were the following: written in English; the participants were under 18 years at baseline; longitudinal, prospective studies; a group of ADHD population at baseline and at follow-up; and suicide thought and behavior as a primary outcome. The exclusion criteria were if the study did not contain empirical data and reviews/meta-analyses and studies which aimed to investigate the drug treatment efficacy of ADHD. Using the inclusion and the exclusion criteria, we screened the titles and the abstracts of the articles found with the search terms. After this initial screening, the relevant full texts of the papers that passed were read, and the ones that met the inclusion criteria were collected. The title, abstract, and full text of the eligible studies were measured by the first author, a process which was carefully reviewed and supervised by the second author, i.e., all included and excluded articles were double-checked according to the inclusion and the exclusion criteria. In case of disagreement, the paper was carefully read again by both authors. The final selection of the relevant studies was based on the agreement of the two authors. The reference lists of the retrieved papers were screened, and papers that possibly met the inclusion criteria were retrieved and studied as well. After the systematic search method, the current literature was screened continuously for eligible studies to include in the review. The reporting of this systematic descriptive review follows the PRISMA statements. Due to the clinical and methodological heterogeneity of the included studies, a narrative approach to data synthesis and presentation was undertaken. To assess the methodology quality of the selected cohort studies, we used the Newcastle–Ottawa Scale Cohort Studies supplement (19), which was based on the agreement of the two authors.

## Results

**Figure 1:**
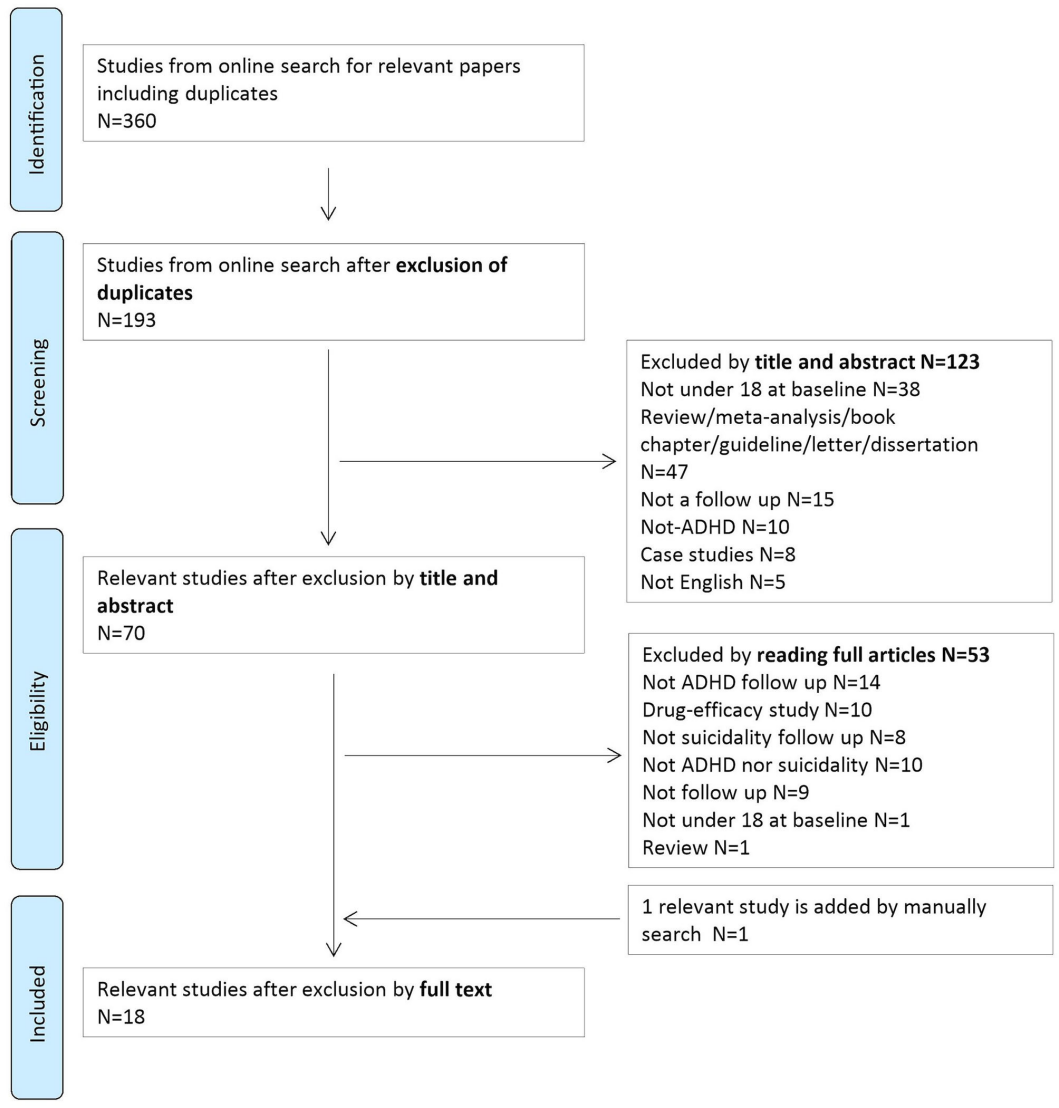
PRISMA flowchart of the selection method.

After the systematic search, we found 360 articles in total, including duplicates. Additionally, one article was included manually (20). After the screening process, 18 papers were finally included in the systematic review (10, 20–36) (Table 1). The selection process is summarized in the PRISMA flowchart (Figure 1).

**Table 1:** Included relevant articles examining associations between ADHD and suicidal thought and behavior in a follow-up design.

**References**	**Title**	**Year**	**Country**	**Sample**	**Population at baseline**	**Age of the population at baseline**	**Follow-up period (follow-up visits)**	**ADHD assessment**	**Measure of Comorbid conditions**	**Suicide outcome measure**	**Main findings**
Barbaresi et al. (21)	Mortality, ADHD, and psychosocial adversity in adults with childhood ADHD: a prospective study	2013	USA	Birth cohort	ADHD group: *N* = 232 (male ratio: 71.9%); control group: *N* = 335 (male ratio: 62.6%)	From birth; mean = 10.4 years (SD = n.a.) (when research diagnostical criteria were met)	n.a., mean age of the followed-up population = 30.0 years (SD = 1.9)	ADHD in childhood: medical and school records; positive ADHD questionnaire results; documented clinical diagnosis of ADHD; M.I.N.I ‹18 years	M.I.N.I.	Based on medical records	In the ADHD group, significantly more suicide deaths were reported than in the control group
Biederman et al. (22)	New insights into the comorbidity between ADHD and major depression in adolescent and young adult females	2008	USA	Clinical sample	ADHD female group *N* = 123 (ADHD only *N* = 43; ADHD + MD, *N* = 80), non-ADHD female group: *N* = 112 (controls, *N* = 89; controls + MD, *N* = 23)	Female subjects ages 6–18 years; control mean = 11.7 (SD = 2.8); control + MD mean=13.9 (SD =2.4); ADHD only MD mean = 10.0 8 (SD =3.3); ADHD + MD mean = 12.1 (SD = 3.2)	Follow-up: 5 years	ADHD/ MDD: K-SADS-PL ›18 years and the SCID ‹18 years	ADHD/MDD: K-SADS-PL ›18 years and the SCID ‹18 years	Not detailed	After the symptom-level analysis, the ADHD group and the control group only differed significantly in the rates of suicidal thoughts
Caye et al. (10)	Attention deficit/hyperactivity disorder trajectories from childhood to young adulthood: evidence from a birth cohort supporting a late-onset syndrome	2016	Brazil	Community	Childhood ADHD group: *N* = 393 (male ratio: 63.9%); non-ADHD group: *N* = 4,033 (male ratio: 47.9%)	11 years of age (SD = n.a.)	7 years (follow-up: 18 to 19 years of age)	Baseline: SDQ (parent and self-report); follow-up: ADHD, ASRS	M.I.N.I.	M.I.N.I—MDD supplement	Significantly higher number of self-reported suicide attempts in children and young adults with ADHD than without, what remained after excluding comorbidities
Chronis-Tuscano et al. (23)	Very early predictors of adolescent depression and suicide attempts in children with attention deficit/hyperactivity disorder	2010	USA	Clinical sample	ADHD group: *N* = 125 (male ratio: 85.6%), non-ADHD group: *N* = 123 (male ratio: 81.3%)	ADHD group: age 4–6 years (mean: 5.2 years; SD = 0.7); ADHD group: 4–6 years of age (mean: 5.2 years; SD = 0.8)	Approximately annual assessments during follow-up year 1 through 4, 6 through 9, and 12 through 14	DISC (for parents); Impairment Rating Scale (for teacher)	DISC for children (for parents); follow-up year 6 through 14 DISC for children (for youth)	Annually at follow-up, year 6 through 14: DISC (for parents and children)	The baseline 4–6 years ADHD group showed a greater risk for suicide attemps and emergence of MDD through reaching year 18 than comparisons. The risk for concrete suicide plans was higher in girls and participants with baseline ADHD-C subtype and baseline ADHD-C and ADHD-HI subtypes for suicide attempts
Forte et al. (20)	Developmental trajectories of childhood symptoms of hyperactivity/inattention and suicidal behavior during adolescence	2020	Italy	Community	Community group, *N* = 1407 (male ratio = 47.2%)	5 months (mean age = n.a.)	Follow-up: 17 years (annually or bi-yearly assessment)	6, 7, 8, 10, and 12 years of age: BQ (for teacher)	Centre for Epidemiological Study Depression Scale; children depression symptoms (for teacher)	13, 15, and 17 years of age: item: “In the past 12 months, did you ever seriously think of attempting suicide?”, if yes: “In the past 12 months, how many times did you attempt suicide?”	In boys, the high and the moderate ADHD trajectory groups showed a greater risk than the low-trectory group for suicide ideation; only high-trajectory group *vs* low-trajectory group was significant for suicide attempt. In girls, there were no significant differences among the trajectory groups
Galera et al. (24)	Hyperactivity–inattention symptoms in childhood and suicidal behaviors in adolescence: the Youth Gazel Cohort	2008	France	Community cohort	Total *N* = 916 (male ratio: 46.0%)	4–18 years (mean = n.a.; SD = n.a.)	Follow-up: 8 years	Baseline: CBCL (for parents); follow-up: a checklist of problems (derived from the CBCL and adapted for young adults)	CBCL	Follow-up: detailed questions about suicidal thought and behaviors (estimate lifetime, previous 12 months and 30 days)	Males with hyperactivity– inattention symptoms showed a significant association with lifetime suicide plans or attempts and nearly significant with 12-month plans/attempts. The association was not significant in the female group with hyperactivity–inattention symptoms. The association was not significant between any subgroups and lifetime and 12-month suicide ideation. Participants with childhood hyperactivity– inattention subtype had a greater risk for lifetime suicide plans/attempts
Goldston et al. (25)	Psychiatric diagnoses as contemporaneous risk factors for suicide attempts among adolescents and young adults: developmental changes	2009	USA	Clinical sample	Total *N* = 180 (male ratio: 49.4%), ADHD subgroup *N* = 29 (male ratio: n.a.)	12–18 years; mean = 14.8 years (SD = 1.6; 12.0–18.4)	Mean = 10,8 years (SD = 3.4); mean number of assessments for active participants: 12.8 (SD = 4.0)	Baseline: ISCA; follow-up: FISA	Baseline: ISCA; follow-up: FISA	Baseline: ISCA; follow-up: FISA	There was a significant relationship between simultaneous diagnosis (MDD, dysthimic disorder, GAD, panic disorder, ADHD, CD, SUD), including ADHD and the future suicide attempts. Strengthened relationship was observed to co-occur with psychiatric disorders (MDD, GAD, ADHD, SUD) and suicide attempts when the participants became older
Gordon and Hinshaw (26)	Parenting stress as a mediator between childhood ADHD and early adult female outcomes	2017	USA	Clinical cohort	ADHD female group: *N* = 120, control female group: *N* = 81	ADHD female group: 6–12 years of age (Mean = 9.7; SD = 1.7), simply Hyperactivity ADHD type excluded; Control female group: 6–12 years of age (Mean = 9.3, SD = 1.6)	10 years (follow-up 1: 5 years, follow-up 2: 10 years)	Baseline: DISC-IV, CBCL, CDI (for parent)	Baseline: DISC-IV, CBCL, CDI (for parent), CDI, assessing maltreatment; follow-up 1: CBCL (for parent), CDI ‹18 years, ABCL, ASR, BDI ›18 years, Self-Injury Questionnaire; follow-up 2: CBCL (for parent) self-reported CDI ‹18 years, ABCL, ASR, BDI ›18 years, modified version of the Self-Injury Questionnaire	Follow-up 1, 2: The Barkley Suicide Questionnaire, FIP	In the female ADHD group, there were more suicide attempts than in the female controls (not detailed). The mother–daughter relationship showed a mediator role between childhood ADHD and young adult NSSI and an indirect effect between childhood ADHD and young adult depressive symptoms
Guendelman et al. (27)	Early-adult correlates of maltreatment in girls with attention deficit/hyperactivity disorder: increased risk for internalizing symptoms and suicidality	2016	USA	Clinical cohort	ADHD female group: *N* = 120 (ADHD-C, *N* = 93; ADHD-IT, *N* = 47)	ADHD female group: 6-12 years of age (Mean = 9.6; SD = 1.7), simply Hyperactivity ADHD type excluded	**10** years (follow-up 1: 5 years, follow-up 2: 10 years)	Baseline (for parents): DISC-IV	Baseline: DISC-IV, CBCL, CDI, SNAP-IV (for parent), CDI, assesing maltreatment; follow-up 1: CBCL (for parent), CDI ‹18 years, ABCL, ASR, BDI ›18 years EDI, EAT-26, WIAT-II, Self- Perception Profile for Adolescents, Self- Perception Profile for Adolescents, Self-Injury Questionnaire; follow-up 2: CBCL (for parent) Self-Reported CDI ‹18 years, ABCL, ASR, BDI › 18 years, modified version of the Self-Injury Questionnaire	Follow-up 1, 2: The Barkley Suicide Questionnaire, FIP	ADHD females with maltreatment experience before showed a greater number of suicide attempts (33%) than non-maltreated ADHD females (13%)
Huang et al. (28)	Risk of suicide attempts in adolescents and young adults with attention deficit–hyperactivity disorder: a nationwide longitudinal study	2018	Taiwan	Register-based	ADHD group: *N* = 20,574 (male ratio: 78.8%), control group: *N* = 61,722 (male ratio: 78.8%)	Adolescents and young adults with ADHD Mean = 14.94 years (SD = 4.46) years, Controls Mean = 14.94 years (SD = 3.49)	Follow-up: 2-11 years; baseline: 01 January 2001–31 December 2009; follow-up: from baseline to 31 December 2011	Diagnosis of ADHD and psychiatric comorbidities was made by board-certified psychiatrists	Based on medical records	Based on medical records	The incidence of first and repeated suicide and the probability of suicide attempts were significanty higher in the ADHD group than in the control group, and the participants with ADHD were younger at first attempt. Medical treatment did not increase the risk of first/repeated suicide attempts. Long-term methylphenidate treatment showed a decreased risk of repeated suicide attempts in men
Hurtig et al. (36)	Suicidal and self-harm behavior associated with adolescent attention deficit–hyperactivity disorder—a study in the Northern Finland Birth Cohort 1986	2011	Finland	Birth cohort	ADHD group: *N* = 188, control group: *N* = 169 (male ratio: n.a.)	Participants from the same cohort	16 years (follow-up 1: age 7 to 8 years, follow-up 2: 16 years)	Screening of ADHD: SWAN; defining the clinical diagnosis: K-SADS-PL	K-SADS-PL	K-SADS-PL	The ADHD group showed a greater number of suicidal ideation, and the ADHD diagnosis had a strong effect of suicidal ideation after controlling other predictors. Female gender, comorbid depression and anxiety, and childhood emotional and behavioral problems were also associated with suicidal thoughts
Lan et al. (29)	Comorbidity of ADHD and suicide attempts among adolescents and young adults with bipolar disorder: a nationwide longitudinal study	2015	Taiwan	Register-based	ADHD + BP group: *N* = 500 (male ratio: 66.2%), BP only: *N* = 1,500 (male ratio 66.2%)	15–24 years; Group with Bipolar disorder and ADHD Mean=19.11 years (SD = 2.84); Group with Bipolar disorder Mean = 19.11 years (SD = 2.84)	Follow-up: 3–10 years; baseline: 01 January 2002–31 December 2008; follow-up: from baseline to 31 December 2011	Diagnosis of ADHD and psychiatric comorbidities was made by board-certified psychiatrists	Diagnosis based on the ICD-9-CM	Based on medical records	Bipolar adolescents and young adults with ADHD co-occurence associated a greater incidence of attempted suicide than the bipolar only group. After adjustment, ADHD was an independent risk factor for lifetime-attempted suicide later among adolescents and young adults with bipolar disorder
Ljung et al. (30)	Common etiological factors of attention deficit/hyperactivity disorder and suicidal behavior: a population-based study in Sweden	2014	Sweden	Register-based	ADHD group: *N* = 51,707 (male ratio: 69.8%), control group: *N* = 258,535 (male ratio: 69.8%)	ADHD group: Age 3-40 years; Control group: matched 1:5 on sex and birth year.	n.a. (lifetime follow-up to age 40 years)	Register-based medical records	Based on medical records	Register-based data (suicidal behavior was allowed at age 12 years or older)	The group with ADHD diagnosis showed a greater risk for suicide attempt and suicide death after adjusting for comorbid psychiatric disorder. High risk of suicide attempts and suicide deaths was observed in first-degree relatives (parents, full siblings)
Miller et al. (31)	Childhood executive function continues to predict outcomes in young adult females with and without childhood-diagnosed ADHD	2012	USA	Clinical cohort	ADHD female group: *N* = 140 (ADHD-C, *N* = 93; ADHD-IT, *N* = 47), control female group: *N* = 88	ADHD female group: 6–12 years of age, simply hyperactivity ADHD-type excluded; control female group: 6–12 years of age (overall mean age = 9.6) (SD = n.a.); comparison sample matched on age and ethnicity	10 years (follow-up 1: 5 years, follow-up 2: 10 years)	Baseline: DISC-IV (for parent)	n.a.	The number of suicide attempts and the number of NSSI “episodes” that had occurred since their last visit were summed up	Elevated number of suicide attempts/NSSI was observed in the ADHD group compared to controls (only descriptive data were available)
Owens et al. (32)	Girls with childhood ADHD as adults: cross-domain outcomes by diagnostic persistence	2017	USA	Clinical cohort	ADHD female group: *N* = 140, control female group: *N* = 88	ADHD female group: 6–12 years of age; control female group: 6–12 years of age, overall mean age = 9.6 years (SD = 1.7); comparison sample matched on age and ethnicity	16 years (follow-up 1: 5 years, follow-up 2: 10 years, follow-up 3: 16 years)	Baseline: DISC-IV (for parent)	DISC-IV; BDI-II; SUQ; Self-Injury Questionnaire (follow-up 1, 2), follow-up 3: ABCL, ASR, SITBI (since follow-up 2)	Follow-up 1, 2, 3: The Barkley Suicide Questionnaire	The view of a persistent ADHD diagnosis through time as partial and persistent; ADHD females showed greater suicide attempts than comparisons after adjusting for covariates
Strandheim et al. (33)	Risk factors for suicidal thoughts in adolescence—a prospective cohort study: the Young-HUNT study	2014	Sweden	Community	Two-sample group: Young-HUNT1 (recruited 1995–1997) and Young-HUNT2 (recruited 2000–2001), *N* = 2399 (male ratio: 46.5%), attention problems subgroup: *N* = 448 (male ratio: 41.9%)	Two sample group: 13–19 years (Young-HUNT1) and 17–19 years (Young-HUNT2)	Mean follow-up time: 3.9 years (SD = n.a.)	ADHD symptoms: 14-item school-adjustment questionnaire	Questionnaire; pain and tension: headache, neck pain, muscle and joint pain, and palpitations during the past 12 months; alcohol use: number of reported alcohol intoxications; sleep disturbances: defined as difficulties in initiating sleep	Follow-up: item “Have you had thoughts about taking your own life?” (yes/no)	At baseline, comorbid conditions, including attention and conduct-associated problems, more than doubled the odds for suicidal thoughts in males and females. The odds decreased after stratifying by gender
Swanson et al. (34)	Pathways to self-harmful behaviors in young women with and without ADHD: a longitudinal examination of mediating factors	2014	USA	Clinical cohort	ADHD female group: *N* = 140 (ADHD-C, *N* = 93; ADHD-IT, *N* = 47), control female group: *N* = 88	ADHD female group: 6–12 years of age; control female group: 6–12 years of age, mean age = 9.1 years (SD = n.a.); comparison sample matched on age and ethnicity	10 years (follow-up 1: 5 years, follow-up 2: 10 years)	Baseline: DISC-IV (for parent)	CBCL, Teacher Report Form, CDI, Self-Injury Questionnaire	Follow-up 1,2: The Barkley Suicide Questionnaire, FIP	The number of suicide attempts was significantly higher in ADHD-C subtype than the comparison group after adjustment. The persistent diagnosis of ADHD showed a higher rate of suicide attempts compared to the comparisons, but not to the transient ADHD participants. Data showed a significant positive correlation between baseline ADHD status and future suicide attempt, where the internalizing symptoms were estimated to play a partial mediator role
Yoshimasu et al. (35)	Psychiatric comorbidities modify the association between childhood ADHD and risk for suicidality: a population-based longitudinal study	2017	USA	Birth cohort	ADHD group: *N* = 232 male ratio: 71.9%; control group, *N* = 335 (male ratio: 62.7%)	From birth (when research diagnostical criteria were met)	n.a., mean age of the folllowed-up ADHD group = 27.0 years (SD = 2.6); mean ager of the followed-up non-ADHD group = 28.6 (SD = 2.2)	Mini International Neuropsychiatric Interview (M.I.N.I.)	M.I.N.I.	M.I.N.I.—Suicide thoughts and attempt section	A direct effect was identified between childhood ADHD and the criteria for suicidality. GAD had a significant synergic interaction effect for future suicide attempts in ADHD cases

*ABCL, Adult Behavior Checklist; ADHD-C, ADHD combined type; ADHD-IT, ADHD inattentive type; ADHD, Attention Deficit Hyperactivity Disorder; ASR, Adult Self-Report; ASRS, World Health Organization Adult ADHD Self-Report Scale Screener; BDI-II, Beck Depression Inventory-II; BQ, Behavior Questionnaire; C, control; CBCL, Child Behavior Checklist; CD, Conduct Disorder; CDI, Children's Depression Inventory; DISC, Diagnostic Interview Schedule for Children; EAT-26, Eating Attitudes Test; EDI-2, Eating Disorders Inventory; FIP, Family Information Packet; FISA, Interview Schedule for Adults; ICD-9-CM, International Classification of Diseases, Ninth Revision, Clinical Modification; ISCA, Interview Schedule for Children and Adolescents; K-SADS-PL, Affective Disorders and Schizophrenia for School-age Children-Epidemiologic Version; M.I.N.I., Mini International Neuropsychiatric Interview; MD, major depression; SCID, Structured Clinical Interview for DSM-IV; SCL-90R, Symptom Check-List 90R; SITBI, Self-Injurious Thoughts and Behaviors Interview; SNAP, Swanson, Nolan, and Pelham rating scale; SUD, Substance Use Disorder; SUQ, Substance Use Questionnaire; SWAN, Strengths and Weaknesses of ADHD Symptoms and Normal Behaviors; WIAT-II, Wechsler Individual Achievement Test, Second Version*.

The 18 relevant articles (10, 20–36) are listed in Table 1. The included studies differ on several characteristics, such as the study populations, *e*.*g*., the age range of the study population, the study design, e.g., the length of the follow-up period, and the measures of ADHD and suicidality. The quality assessment of the selected studies are listed in Table 2. The paper of Galéra et al. (24) and Goldston et al. (25) were not included to the cohort quality assessment due to different methodological study design.

**Table 2:** Methodological quality assessment of the selected studies-based on Newcastle-Ottawa Scale. In this scale each item could be awarded with a star per section. The number of the stars means the number of the awarded items.

**References**	**Selection**	**Comparability**	**Outcome**
Barbaresi et al. (21)	***	**	***
Biederman et al. (22)	***	**	**
Caye et al. (10)	***	*	*
Chronis-Tuscano et al. (23)	***	**	**
Gordon and Hinshaw (26)	***	Not available^a^	**
Guendelman et al. (27)	***	**	**
Huang et al. (28)	***	**	***
Hurtig et al. (36)	**	**	**
Lan et al. (29)	****	**	***
Ljung et al. (30)	***	**	***
Miller et al. (31)	***	**	**
Owens et al. (32)	***	**	**
Strandheim et al. (33)	**	**	*
Swanson et al. (34)	***	**	**
Yoshimasu et al. (35)	***	**	**
Forte et al. (20)	**	**	*

a *Only descriptive data were available for the suicide attempts (outcome)*.

### Descriptive Analysis of the Included Studies

Based on geographical distribution, the articles investigated the following populations: 10 articles are from North America (21–23, 25–27, 31, 32, 34, 35), five are from Europe (20, 24, 30, 33, 36), two are from Asia (28, 29), and one is from South America (10).

Highlighting the significance of the topic, 11 of the investigated 18 articles were altogether published in the last 5 years (10, 20, 26–30, 32–35).

Eight papers reported data from a clinical sample, constituting the majority of the collected studies (22, 23, 25–27, 31, 32, 34); however, among these eight papers, five (26, 27, 31, 32, 34) were analyses from the same study population [for additional details, see Hinshaw et al. (37), Barbaresi et al. (21), and Yoshimasu et al. (35) who used data from the Rochester Epidemiology Project, Minnesota] (21, 35, 38). Although three studies used register-based data (28–30), among these studies, Huang et al. (28) and Lan et al. (29) used register-based data from Taiwan. Three studies recruited participants from a community sample (10, 24, 33), and birth cohort data were used by three studies (21, 35, 36).

Ten studies enrolled children aged under 12 years at baseline (10, 20, 23, 26, 27, 31, 32, 34–36). However, Barbaresi et al. (21) and Yoshimasu et al. (35) used birth cohort data, and there were no strict age-based inclusion criteria. The baseline age was calculated when the participants met the research diagnostic category; the mean age of the participants was 10.4 years (SD = n.a.) (21, 35). Huang et al. (28), Lan et al. (29), Ljung et al. (30), and Strandheim et al. (33) used a wide age range criterion including child, adolescent, and adult participants (28–30, 33).

Regarding gender distribution in the included studies, 12 studies recruited both male and female participants (10, 20, 21, 23–25, 28–30, 33, 35, 36); among them, eight studies investigated mainly male populations (more than 60% males in the population) (21, 23, 28–30, 35, 36), and four studies demonstrated an approximately balanced male–female ratio population (21, 23, 26). Six studies included only female participants (22, 26, 27, 31, 32, 34).

### Follow-Up Period of the Included Studies

The follow-up period of the included studies varied in a wide range. Seven studies published data after more than a 10-years follow-up period (20, 21, 25, 30, 32, 35, 36). From 5 to 10 years of follow-up data were investigated by 10 studies (10, 22–24, 26–29, 31, 34). The mean follow-up period was <5 years in one study (33).

Nine of the 18 studies planned an exact number of follow-up visits (10, 22, 24, 26, 27, 31, 32, 34, 36). One follow-up visit was planned by Biedermann et al. (22), Caye et al. (10), and Galéra et al. (24). Hurtig et al. (36), Gordon and Hinshaw (26), Guendelman et al. (27), Miller et al. (31), and Swanson (34) planned two follow-up visits; three follow-up visits were accomplished by Owens et al. (32). The average number of research assessments for active participants was 12.8 (SD = 4.0) in the study of Goldston et al. (25).

The three register-based studies, the design of the studies, did not apply an exact number of follow-up visits where data were derived from (28–30).

### Assessment of ADHD

The majority of the studies used structured interviews or medical records to establish the ADHD diagnosis. From these, 11 studies (22, 23, 25–29, 31, 32, 34, 36) used structured interview, and three of the studies used medical data to identify the participants with ADHD diagnosis (21, 30, 35). It was noted that four of the selected articles used a non-diagnostic tool to evaluate the hyperactivity–inattention trajectories and symptoms in the selected population (10, 20, 24, 33).

The NIMH Diagnostic Interview Schedule for Children (DISC) (39) was used to establish the ADHD diagnosis in six of the selected studies (23, 26, 27, 31, 32, 34). Four of these studies used DISC Version IV (DISC-IV) (40). DISC-IV is a highly structured interview addressed to evaluate more than 30 mental disorders, including ADHD. It was designed to be used in large-scale, epidemiological studies (40) using the Diagnostic and Statistical Manual of Mental Disorders IV (DSM-IV) (2) and the International Classification of Disorders (ICD-10) (41). In five studies (26, 27, 31, 32, 34), the ADHD diagnosis was based on the parents' responses about the unmedicated behavior of the participant girls at baseline evaluation (37). Chronis-Tuscano et al. (23) interviewed the mothers or the primary caregivers.

Barbaresi et al. (21) and Yoshimasu et al. (35) investigated a birth cohort. Enrollment to the baseline population, based on the documented school and medical records of the behavior symptoms of the participant, included the ADHD criteria of DSM-IV (2), (42).

Biederman et al. (22) collected a clinical sample based on medical records, but they used the Affective Disorders and Schizophrenia for School-Age Children—Epidemiologic Version (Kiddie-SADS-PL) (43). Kiddie-SADS-PL is a structured, diagnostic tool based on the diagnostic criteria of DSM-IV-TR diagnoses (2). The disorder was positive when either the participant's or his/her mother's information met the diagnostic criteria (22).

Goldston et al. (25) applied the Interview Schedule for Children and Adolescents (ISCA) (44) at baseline for children and the adult version of ISCA, the Follow-Up Interview Schedule for Adults (FISA) (45), at the follow-up phase. Both the ISCA and the FISA are highly structured interviews to estimate the severity, duration, and functional impairment of the symptoms (44, 45).

Ljung et al. (30) identified the ADHD cases from register-based medical records.

Hurtig et al. (36) used a two-step identifying procedure for ADHD participants. They used the parents version of the Strengths and Weaknesses of ADHD Symptoms and Normal Behaviors (SWAN) (46) to evaluate the continuum from attention problems to attention skills with a seven-point rating scale. After screening, the ADHD diagnosis was defined by Kiddie-SADS-PL (43). The ADHD diagnoses were made by board-certified psychiatrists based on their clinical interview and judgement in two studies (28, 29).

In the study by Forte et al., the teacher version of the Behavior Questionnaire (BQ) was used to evaluate ADHD symptoms. The BQ was developed with the incorporation of items from the Child Behavior Checklist (CBCL) (47), the Ontario Child Health Study Scales (48), and the Pre- school Behavior Questionnaire (49). The BQ contains four items for hyperactivity and three items for inattention symptoms.

Galéra et al. (24) used the CBCL (50) for the measurement of ADHD symptoms. CBCL is a self-reported questionnaire that mainly measures the internalizing and the externalizing dimensions, including attention problems (50). Caye et al. (10) used parent and self-report data of the Strengths and Difficulties Questionnaire (SDQ) (51, 52). The SDQ is a 25-item self-report questionnaire that evaluates behavioral problems and prosociality (51, 52). To define the child ADHD cases, they used a threshold of eight points associated with impairment (10). The SDQ contains four problem scales: emotional problems, conduct problems, hyperactivity problems, and peer problems (52). Strandheim et al. (33) assessed the participants with a 14-item school-adjustment questionnaire to evaluate attention and conduct symptoms.

### Measurements of Suicidality

The assessment of suicidality focused on suicidal thoughts, suicidal attempts, or both. Two articles investigated suicidal thoughts (22, 33), six studies assessed only suicidal attempts (10, 21, 28–31), and nine studies investigated both phenomena of suicidal thought and behavior (20, 23–27, 32, 34, 35).

#### Suicidal Thought

Only two studies investigated on suicide thoughts exclusively. Strandheim et al. (33) used only one screening question (“Have you had thoughts about taking your own life?”) to identify the participants with suicide thoughts at the follow-up visit. However, the article by Biederman et al. (22) did not detail the measurements of suicidal thoughts; they used the Kiddie-SADS (43). The suicide ideation and behavior screening interview contains items about thoughts of death, suicidal ideation, presence of suicide attempts, non-suicidal self-harming behavior, and medical lethality and intent associated with a possible suicide attempt (43).

#### Suicide Attempts

Majority of the selected articles, which investigated only suicide attempts, obtained data from medical records (21, 28–30).

Huang et al. (28) and Lan et al. (29) used a Taiwanese National Health Insurance Research database which includes demographic data, date of visit, disease diagnoses, and medical interventions (28).

By contrast, Barbaresi et al. (21) used medical records to identify the mortality of the participants, including suicide attempts. The infrastructure and the medical data of the study were based on the Rochester Epidemiology Project (38).

In another study, several Swedish nationwide registers were used in the research of Ljung et al. (30); among these, the Swedish National Patient Register (53) was used for the medical records and the Swedish cause of death register (54) to identify the causes of deaths.

Beyond the medical record-based studies, Caye et al. (10) investigated suicide attempts at age 11 years as part of the Pelotas (Brazil) Birth Cohort Study (55) and at 18 age of years as part of the assessment of Major Depression Disorder (MDD), which included the Mini International Neuropsychiatric Interview MDD supplement (56). It was noted that this measurement was based on a self-report setting.

Miller et al. (31) did not calculate an independent number of suicide attempts but created a non-suicidal self-injury (NSSI)/suicide attempt count by summing up the number of NSSI and suicide attempts since the last visit. The questionnaire which measured the suicide attempts was not detailed in the article.

#### Suicide Thoughts and Suicide Attempts

Gordon and Hinshaw (26), Guendelman et al. (27), Owens et al. (32), and Swanson et al. (34) used the Barkley Suicide Questionnaire (57). This is a self-reported questionnaire which includes the following three questions: “Have you ever attempted suicide?,” “Have you ever considered suicide?,” and “Have you ever been hospitalized for an attempt?” to evaluate suicide thoughts and attempts. Additionally, the Family Information Profile was used in the follow-up phase, which contains a suicide attempt sub-questionnaire reported by caregivers.

Chronis-Tuscano et al. (23) used the DISC-IV (40) to evaluate suicide attempts at the assessment in year 9 of the follow-up. Through the 6–14 years of the follow-up period, at every annual assessment, the participants and the parents were asked to report suicide attempts or suicidal ideation in the previous 6 months.

In the study of Forte et al. (20), a two-step questionnaire was used to detect suicidal thought and attempts: “In the past 12 months, did you ever seriously think of attempting suicide?” and “In the past 12 months, how many times did you attempt suicide?” if the first answer was positive.

Hurtig et al. (36) evaluated suicidal thought and behavior by the mood disorders section of K-SADS-PL (43), which includes five items for measuring suicidality and self-harm. To maximize the sample size, the authors included “recurrent thoughts of death” and “suicidal ideation” and the items “suicidal acts—medical lethality” and “non-suicidal physical self-damaging acts.”

In the study of Galera et al. (24), information was collected from the participants about suicide thoughts or attempts in the follow-up phase. They used a detailed questionnaire about the presence and the frequency of suicidal ideations, suicide plans, and suicide attempts in the previous 30 days/12 months and lifetime.

Goldston et al. (25) investigated suicide attempts, thoughts, and behaviors at baseline and the follow-up period. Suicide attempts, thoughts, and behaviors were assessed by the ISCA (44) interview, and additional information was obtained from treatment/medical records and the parents about the suicide attempts at baseline. In the follow-up period, the ISCA (44) and the FISA (44, 45) were used to evaluate suicide attempts, thoughts, and behaviors since the last visit. Additional information about the dates of suicide attempts was collected from the medical treatment, school, and legal records. In the follow-up period, they used the Lethality of Suicide Attempt Rating Scale (58) to rate the medical lethality of the suicide attempt using a 0–10 scale.

Yoshimasu et al. (35) used the Mini International Neuropsychiatric Interview (59) to evaluate suicide behavior (thoughts and plan) and attempts. Five items rate the suicide thoughts and plan in the previous 1 month, and one item is about the lifetime suicide attempts. They created groups based on the severity of the suicide questions as none (0), low (1–5), moderate (6–9), or high (10+) risk groups (35) where 10 points was calculated in the case of suicide attempts in the last month.

### Long-Term Association Between ADHD and Suicidality

Although the number of follow-up visits varied in a wide range among the selected studies, the vast majority of the studies collected data after more than a 5-years follow-up period.

Firstly, we present the articles which investigated the long-term risk of suicidal thoughts (22, 33), after that those ones which investigated suicidal attempts (10, 21, 28–31), and finally studies which measured the presence of both phenomena in children and adolescents with ADHD (23–27, 32, 34–36).

#### Long-Term Association Between ADHD and Suicidal Thoughts

Strandheim et al. (33) followed up an adolescent cohort population to investigate the presence of suicide thoughts, where 18.7% of the population showed attention problems (*N* = 448). After 17 years of follow-up, the gender-adjusted odds ratio was 1.3 (CI = 1.0–1.7). This odds ratio was lower compared to the anxiety/depressive, conduct problems, and insomnia subgroups.

In an adolescent and young adults female sample, Biedermann et al. (22) investigated the association between ADHD and major depression (MD). They measured the suicide thoughts in four groups: control, ADHD only, MD only, and ADHD + MD. After analyses of the symptom level, there was a significant difference between the ADHD only group and the control group in the rates of suicidal thoughts (68 vs. 43%, respectively; *p* < 0.05).

#### Long-Term Association Between ADHD and Suicidal Attempts

In a large birth cohort study, Barbaresi et al. (21) focused on the mortality and psychosocial functioning of the adult ADHD population who were diagnosed with ADHD in childhood. Among the mortality rates, there was a significant difference between the number of suicide deaths of the ADHD individuals (*N* = 3 from 367) and the controls (*N* = 5 from 4,946) (standardized mortality ratios for suicide = 4.83, 95% CI, 1.14–20.46; *p* = 0.032).

Another birth cohort study by Caye et al. (10) followed up children with ADHD and controls for more than 7 years. A significantly higher number of self-reported suicide attempts was found in children with ADHD than those without [35 (10%) vs. 213 (6%), *p* = 0.003] and in young adults with ADHD than in young adults without ADHD [75 (15.2%) vs. 180 (5.1%), *p* < 0.001).

Huang et al. (28) focused on the suicide attempts as well of ADHD adolescent patients and age- and gender-matched controls in a population-based, longitudinal cohort study, where the data were derived from health insurance records. The incidences of a first and repeated suicide attempts were significantly higher (0.5 and 0.1%, respectively, both *p* < 0.001) in the ADHD group than in the controls, similarly to the results of Caye et al. (10). At their first suicide attempt, the ADHD patients were significantly younger (19.35 vs. 20.77 years, *p* < 0.001) than the control group. The ADHD patients showed a higher probability to attempt suicide (*p* < 0.001), and the data showed that the ADHD diagnosis was an independent risk factor for any suicide attempt (hazard ratio = 3.84, 95% CI = 3.19–4.62) and repeated suicide act (hazard ratio = 6.52, 95% CI = 4.46–9.53) regardless of the gender and the age subgroup. An interesting finding was that long-term methylphenidate (MPH) treatment showed a significant association with a decreased risk of repeated suicide attempts in the case of ADHD men (hazard ratio = 0.46, 95% CI = 0.22–0.97). By contrast, MPH and atomoxetine did not show a significant association with the first suicide attempts and the repeated suicide attempts.

With a sophisticated register-based design, Lan et al. (29) focused on adolescents and young adults with bipolar disorder and a comorbidity of ADHD. They found the ADHD diagnosis as an independent risk factor for suicide after further adjustment for anxiety disorder (HR: 2.82, 95% CI: 1.38–5.77) and adjustment for disruptive behavior disorders, alcohol use disorders, and substance use disorders (HR: 2.38, 95% CI: 1.13–5.00).

Another register-based study (which used patient and prescribed drug register data) by Ljung et al. (30) assessed the genetic and environmental risk factors of patients with ADHD and their relatives for suicide attempts and completed suicide. They found suicide attempts of 0.2 vs. 0.02% who had completed suicide, which meant a relatively high risk for suicide attempts [OR = 3.62 (95% CI, 3.29–3.98)] and completed suicide [OR = 5.91 (95% CI, 2.45–14.27)] among patients with ADHD, and this risk remained after adjusting for comorbid disorders.

Miller et al. (31) followed-up pre-adolescent girls with and without ADHD for 10 years and collected data about the number of suicide attempts and the NSSI which were summed for analysis. Limiting the evaluability of the results, only descriptive data were published with regard to the number of suicide attempts. The mean continuous number of the follow-up suicide attempts and NSSI in the comparison group (*n* = 71–86) were 0.38 (range 0–8; SD = 1.24) and 1.12 (range 0–12; SD = 2.33) in the ADHD group (*n* = 107–137).

Goldston et al. (25) focused on the relationship between suicide attempts and psychiatric disorders, including ADHD, in an adolescent population who were followed up for 13 years after recruitment following an inpatient admission. Altogether 14.3% of the first suicide attempters and 25.0% of repeated suicide attempters had a diagnosis of ADHD. From adolescence through young adulthood, patients with ADHD showed a greater risk for suicide attempts (χ^2^ = 6.91, *p* = 0.009; hazard ratio 2.41). For a broader view, the most prominent risk for suicide attempts was shown by patients with MD, panic disorder, conduct disorder, and substance use. It was noted that, in the multivariate model, the association in the case of ADHD did not remain significant (χ^2^ = 1.53, *p* = 0.216; hazard ratio 1.52). A very interesting finding is that the relationship between ADHD and suicide attempts strengthened with the age of the patients.

#### Long-Term Association Between ADHD and Suicidal Thoughts and Attempts

Regarding those studies, which assessed both suicide thoughts and attempts, Chronis-Tuscano et al. (23) investigated the risk for depression and suicide thought and behavior in children diagnosed with ADHD who were 4–6 years of age and in control participants without ADHD. They followed up the 4–6-year-old children with seven assessments in assessment years 6 to 14 (9–18 years of age). During the follow-up phase, 12.0% of children with baseline ADHD and 1.6% of children without baseline ADHD reported a concrete suicide plan (χ^2^ = 5.38, *p* < 0.03; hazard ratio 5.79). In the case of suicide attempts, 18.4% of patients with ADHD and 5.7% of the control group had a suicide attempt (χ^2^ = 8.26, *p* < 0.005; hazard ratio 3.60). For subgroups, only the combined subtype of baseline ADHD (ADHD-C) had a greater risk for concrete suicide vs. the controls (χ^2^ = 6.52, *p* < 0.02; hazard ratio 7.19); however, the hyperactive–impulsive (ADHD-HI) subtype (*p* < 0.06) and the inattentive (ADHD-IT) subtype (*p* = 0.99) had no greater risk for concrete suicide ideation than the participants without ADHD. Baseline ADHD-C (χ^2^ = 6.42, *p* < 0.02; hazard ratio 3.20) or ADHD-HI (χ^2^ = 4.12, *p* < 0.05; hazard ratio 3.37) also had a greater risk for suicide attempts than the control group. Consistent with the findings of Biedermann et al. (22), children with ADHD diagnosis at ages 4–6 years have a greater risk for MD through adolescence. Additionally, the maternal depression of the group having ADHD at baseline showed a greater risk for concrete suicide ideation during the follow-up (χ^2^ = 9.55, *p* < 0.05; hazard ratio 2.57).

Galéra et al. (24) followed up a 7–18-year-old community sample after 8 years. It was noted that the participants were measured with a hyperactivity symptoms scale; no ADHD diagnostic tool was used. In total, based on a multiple logistic regression model, the childhood HI-s had a greater risk for lifetime suicide plans or attempts (OR = 3.25, 95% CI = 1.26–8.40) and 12-months suicide plans or attempts (OR = 5.46, 95% CI = 1.16–25.81). The correlation was independent of both externalizing and internalizing problems and regular cannabis smoking as well.

As mentioned before, Gordon and Hinshaw (26), Guendelman et al. (27), Owens et al. (32), and Swanson et al. (34) conducted analyses taken from the same study population [for details, see Hinshaw et al. (37)], where the participants were pre-adolescent girls diagnosed with combined and inattentive-type ADHD and assessed without ADHD at baseline while unmedicated.

Gordon and Hinshaw (26) focused on parenting stress as a possible mediator between ADHD and young adult outcome in a 5- and 10-years follow-up setting. There were more suicide attempts in the ADHD group than in the control group after a follow-up [17.1 vs. 5.1%, χ^2^ (1, *N* = 199) = 6.30, *p* < 0.05]. The data assessment did not show a correlation between the number of suicide attempts and parental distress (*p* = 0.13) or stress-inducing dysfunctional parent–child interactions (*p* = 0.11).

Guendelman et al. (27) investigated the role of maltreatment experience (physical abuse, sexual abuse, or neglect) in childhood and/or adolescence in the future outcome of internalizing symptoms and suicidality. The authors found a greater risk for suicide in the ADHD group, where maltreatment was experienced [33 vs. 12.8%; χ^2^ (1, *N* = 124) = 6.59, OR = 1.85, 95% CI = 1.14, 3.00; *p* = 0.01]. Additionally, this group showed a greater impairment in internalizing symptomatology (anxiety and depression), eating disorder symptomatology, and self-esteem. The analyses of covariance (age, socioeconomic status, prenatal risk, adopted, or in foster care, baseline depression/dysthymic disorder, or anxiety disorder) revealed that the difference between maltreated and non-maltreated suicide attempts was significant (Wald = 5.55, *p* = 0.02).

Owens et al. (32) performed a wave three follow-up period to 16 years to evaluate the functional and symptomatological outcome of the female participants diagnosed with ADHD in childhood in 10 domains including self-injury. The baseline ADHD group was divided into three subgroups based on the persistence of the ADHD diagnosis at the follow-up visits: “desisters,” “partial,” and “persisters.” The number of attempted suicides was less frequent among participants in the control (6.1%) and the desister groups (3.4%) than in the partial (23.7%) or the persisters groups (27.4%) (df = 3, 197; *F* = 17.01; *p* < 0.001). In the pairwise comparisons, the partial (OR = 5.0) and the persistent (OR = 5.8) groups showed higher rates in suicide attempt than the comparisons. However, a relatively large odds ratio showed in partial vs. desisters (OR = 9.0) and persisters vs. desisters (OR = 10.6); there was no significant difference because of the small sample size.

Swanson et al. (34) assessed the dates derived from the previously mentioned follow-up population and focused on the possible mediating factors between ADHD and NSSI or suicide attempts. The authors divided the participants in two ways of grouping: firstly, ADHD-C, ADHD-IT, and comparisons; secondly, transient ADHD group, persistent ADHD group, and lifetime non-diagnosed group. The rates of suicide attempts were significantly higher in the ADHD-C group (22%) than in the ADHD-IT group (7%, OR = 3.5) and comparisons (6%, OR = 4.5). In the case of longitudinal grouping, the persistent ADHD group (22%) reported more suicide attempts than the comparison group (4%, OR = 6.7), but not the transient group (13%, OR = 2.0). In the bootstrap mediator analyses, the data showed a significant positive correlation between baseline ADHD status and future suicide attempts [*b* = 0.28, standard error = 13, *t*_(195)_ = 4.12, *p* < 0.001, *R*^2^ = 0.08] and in the 5-years follow-up. The authors reported the internalizing symptoms as a partial mediator between baseline ADHD status and future suicide attempts (indirect effect = 0.11, SE = 0.05, 95% CI = 0.03–0.25).

In another longitudinal study, Hurtig et al. (36) investigated suicide thoughts and acts in a birth cohort population with and without ADHD after a 16-years follow up. In this population, the ADHD diagnosis of the participants was associated with suicidal thoughts (*n* = 53/104 vs. n = 40/169), with 6.1 OR (95% CI 2.34–16.0). On the other hand, the number of suicidal acts was low in both groups, without a significant difference (6/104 vs. 4/169, *p* = 0.855).

Finally, Yoshimasu et al. (35) followed up an ADHD birth cohort through adulthood. They found a significantly higher rate of suicidality in the group of childhood ADHD (22%) than in non-ADHD participants (10.4%) (*p* < 0.001, OR = 2.42; 95% CI = 1.51–3.86).

#### Gender Differences in the Association Between ADHD and Suicidality

Regarding gender differences, we overviewed the included studies which investigated a mixed-gender population.

The objective of the study by Forte et al. (20) focused on the sex differences in the context of suicidality and ADHD symptoms with a 17-years follow-up design. Regarding the symptoms, the authors identified low, moderate, and high ADHD trajectories. After the comparison of the trajectories, only boys showed a prominent risk for suicide ideation (moderate vs. low, OR 4.4, 95% CI 1.3–15.1; high vs. low, OR 3.9, 95% CI 1.0–14.9); however, only higher trajectories showed differences vs. low trajectories regarding suicide attempts (OR 4.5, 95% CI 1.1–17.9).

By contrast to these findings, in the study of Strandheim et al. (33), the odd ratios of having suicide thoughts did not show robust differences between genders (boys aOR = 1.3, 95% CI = 0.8–2.1; girls aOR = 1.2, 95% CI = 0.8 to 1.8) among the ADHD participants; however, the follow-up period was only 4 years, compared with that of the previous study which was 17 years (19).

Chronis-Tuscano et al. (22) reported no gender differences (*p* = 0.17) in the case of a concrete suicide plan, but the girls showed a greater risk (χ^2^ = 3.89, *p* < 0.001; hazard ratio 7.37) for suicide attempt than the boys in the same group at 1-year follow-up period in a Cox modeling method. It was notable that the participants were only 5–7 years old at the first year of follow-up.

In the study of Galéra et al. (23), the HI-s boys showed a higher risk of suicide plans or attempts (*p* = 0.02) in a lifetime period than HI-s girls. Neither in male nor in female groups was there a higher risk of suicide ideation in the lifetime and the 12-months period. It was noted that the authors measured just the presence of hyperactivity symptoms; the participants had no established ADHD diagnosis.

Following was a register-based study of Ljung et al. (29), where female patients showed almost twice as much risk for suicide attempts than males [OR = 5.41 (95% CI, 4.60–6.36) vs. OR = 2.93 (95% CI, 2.60–3.29), χ^2^ = 1,271.0; *p* < 0.001)].

#### Role of Comorbidity in the Association Between ADHD and Suicidality

In our review, we found that four of the selected studies included measures of comorbidities in the data analysis.

Biedermann et al. (22) investigated the association of ADHD and MD in adolescent and young adult females. Females with ADHD had a greater risk for MD than the control group [65% (*n* = 80) vs. 21% (*n* = 23)]. The ADHD group showed more than double the risk (HR = 2.5, 95% CI 1.5–4.2; *p* < 0.01) for lifetime MD than the control group after adjustment for comorbid conditions. Following a symptom-level analysis, the ADHD group had a higher rate of suicidal ideation (68 vs. 43%, respectively; *p* < 0.05). For a broader view, MD combined with ADHD showed a greater duration of MD, with more severe MD-related impairment than in controls with MD only.

In the study by Lan et al. (29), data showed that the patients with ADHD and bipolar disorder had a significantly higher incidence of suicide attempts (3.0 vs. 1.1%, *p* = 0.005) than the group with bipolar disorder. Based on the Kaplan–Meier survival analysis, the risk of suicide attempts is greater (*p* = 0.002) in the group of patients with ADHD and bipolar disorder than in patients with bipolar disorder only.

The effect of the psychiatric comorbidities in relation to childhood ADHD diagnosis and suicide thought and behavior was the objective of the study of Yoshimasu et al. (35). In observing the effect of childhood ADHD on suicidality, they found a significant direct effect between the two phenomena (*p* < 0.001, OR = 2.58, 95% CI = 1.54–4.31). Based on the data, the three most prominent comorbid disorders that have a direct effect as a percent change of the total effect were as follows: MDD (25.7%), hypomanic episode (18.3%), and dysthymia (18.1%). They also calculated an expected joint effect to evaluate the synergic (OR exceed the expected OR) or independent effect of ADHD and other psychiatric comorbidities. The data showed a significant synergic effect between generalized anxiety disorder (expected OR = 4.86, OR = 10.94; 95% CI, 4.97–24.08), hypomanic episode (expected OR = 2.66, OR = 7.40; 95% CI, 3.48–15.77), and substance-related disorder (expected OR = 3.02, OR = 6.62; 95% CI, 3.15–13.91).

Finally, Caye et al., in their birth cohort study, reported a higher rate of comorbidities in young adulthood in those participants who had an ADHD diagnosis in childhood. A significantly higher number of self-reported suicide attempts was found in children with ADHD than those without [35 (10%) vs. 213 (6%), *p* = 0.003] and in young adults with ADHD than in young adults without ADHD [75 (15.2%) vs. 180 (5.1%), *p* < 0.001]. These differences remained significant after excluding the comorbidities from young adults with and without ADHD [17 (6.6%) vs. 101 (3.5%), *p* = 0.01].

## Discussion

To our knowledge, this is the first systematic review on the topic of ADHD and suicidality including studies with follow-up design exclusively. By this method, our objective was to summarize the current literature findings to provide an additional perspective and a better understanding of the long-term cause–effect relationship between ADHD and suicidality.

In spite of the methodological heterogeneity of the included studies, which limits conclusions about the suicide risk of ADHD patients, there have clearly been major advances in the available studies with follow-up design. Based on these studies, there is now strong evidence that there is a positive association between the presence of ADHD diagnosis at baseline and having suicidal thoughts and/or attempts at follow-up visits.

In our systematic review, we could identify 18 studies with follow-up design which investigated the suicidal risk of ADHD patients (10, 20–36). The methodology of the selected studies shows a wide heterogeneity. From the 18 selected studies, 14 used a standardized method to establish the diagnosis of ADHD, including standardized measurement or reliable medical data (21–23, 25–32, 34–36). The other four studies measured ADHD symptoms in a dimensional approach without an established ADHD diagnosis, which limits the interpretation of these results.

For suicide measuring instruments, only half of the studies investigated both suicide thoughts and attempts (20, 23–27, 32, 34, 35). After excluding one study based on medical records, only five studies (10, 23–25, 35) used a detailed questionnaire for identifying suicide thoughts, ideation, or attempts. Suicide behavior can be interpreted along a continuum, as it consists of passive ideation, active intent, and specific plan (60). Measuring these elements together could elevate the interpretability of the data in the future.

Regarding our descriptive findings, the majority of the studies (10/18) are from North America; however, four of them collected data from the same population sample. The selected studies investigated only 10 separate populations. The data were derived from clinical samples (22, 23, 25–27, 31, 32, 34), register-based data (28–30), data from community samples (10, 20, 24, 33), and data from birth cohorts (21, 35, 36); however, the majority of the samples were derived from clinical samples (8 of 18). Five of these eight studies (26, 27, 31, 32, 34) from a clinical population followed up the same study population (37). The two register-based prospective studies assessed children from the same birth cohort (38).

The majority of the studies (12/18) assessed population from both sexes (10, 20, 21, 23–25, 28–30, 33, 35, 36); however, we found a relatively low number of studies that examined a population with balanced gender ratios (10, 20, 24, 25, 33).

Although ADHD is more prevalent among boys than girls, none of the selected studies investigated a male population only; however, one-third (6 of 18) of the studies followed up female participants only (22, 26, 27, 31, 32, 34).

Although the age of the study populations at baseline was mostly (10/18) under 12 years, the included studies covered a wide age range at the final follow-up visits.

Although the higher retention rate in child or adolescent longitudinal studies challenges the long-term design and an eligible sample size, except for one study (33), the followed-up period was longer than 5 years; moreover, the majority of the studies had a 5- to 10-years follow-up period.

In the case of measurement of suicidality, the most assessed domain was suicide attempt. Altogether 14 of the selected 18 articles assessed suicide attempts, of which 13 of them reported a significant association with baseline ADHD symptoms. Despite the fact that, among these studies, 12 used available data from detailed statistical probes, our results highlighted the growing evidence of the elevated risk of ADHD for later suicide attempt.

Regarding suicidal thoughts and plans, six studies published data, of which five reported a significant risk context of the baseline ADHD symptoms. Childhood ADHD diagnosis is deemed to be a significant risk factor for future suicide deaths; however, only a few (21, 30) longitudinal studies in child and adolescent populations are available in the current review on this topic.

These results are consistent with the recently published systematic reviews and meta-analyses, which mainly focused on a wide range of population of adults, adolescents, and children with ADHD and a cross-sectional design, and found a positive correlation between ADHD and suicidality (15, 17).

The selected studies showed a significant range in terms of the heterogeneity of different methodologies. However, the majority of the studies used standardized ADHD diagnostic tools or medical data (21–23, 25–32, 34–36); a majority of the selected articles investigated ADHD symptom severity scales (10, 20, 24, 33). The differences between the interpretation of the categorical and the dimensional symptom estimation could complicate the understanding of the results.

A selective review by Giupponi et al. (18) suggested a correlation between ADHD and an increased risk of suicidal ideation and attempts in adolescent and child populations, although they found a controversial direct connection between ADHD and suicidal thought and behavior (18). The non-systematical review of James et al. (13) focused on psychological autopsy studies and long-term follow-up studies of ADHD children, children, adolescents, and young adults. The data suggest an association between ADHD and completed suicide, especially for younger males. Impey and Heun (16) found that ADHD diagnosis is more frequent in “suicidal groups” than in controls, and suicide thought and behavior were more common in prediagnosed groups than in controls. A current systematic review by Balázs and Keresztény (15) concluded a positive association between ADHD and suicidal thought and behavior in all age groups and in both sexes.

Among the risk factors of suicidality, in this review, we focused on psychiatric comorbidities in two ways: We evaluated the risk for developing comorbid disorders in ADHD population. Two studies (22, 35) reported an elevated risk for the development of MD in ADHD. These data delineate the predictor role of ADHD for future MD and suicide thought and behavior, which is consistent with two other previous reviews (61, 62). These findings suggest some overlap between ADHD and MD and support the possible mediator role of MD between ADHD and suicidality.

However, there are no clear data on the role of comorbidities in suicide in ADHD population. James et al. (13) estimated that having the ADHD diagnosis may increase the risk of suicide in males by increasing the severity of comorbid conditions, particularly conduct disorder and depression. Balázs and Keresztény (15) conclude the mediator role of comorbid conditions between ADHD and suicidality.

Among the selected articles of the current review, two studies investigated the effect of comorbid conditions in suicidal thought and behavior in an ADHD population. Yoshimasu et al. (35) evaluated a direct effect between ADHD and suicidality and other comorbidities, i.e., GAD, hypomanic episode, and substance use disorder had a synergic effect for future suicidality. In contrast, Swanson et al. (34) identified a partial mediator role of the internalizing disorders in the context of suicide in ADHD patients. We should highlight that these two studies differ in the gender ratio, which can be a possible explanation of the co-occurring disorders with ADHD.

A further important topic among the objectives of this review was to investigate the risk of suicide in patents with symptoms of ADHD in the context of sex differences. Previously, Nigg et al. (14) reported an association between ADHD and an elevated risk of suicide attempts (particularly in girls) and lethal suicide attempts (particularly in boys). Among the selected articles of the current review, four studies published data on sex differences in suicide thought and behavior. The results by Ljung et al. (30) showed a nearly doubled risk of suicide attempts for ADHD girls than boys. Forte et al. (20) and Galera et al. (24) also conclude a significant risk for suicide thoughts and attempts only in boys with ADHD symptoms, but there was no established ADHD diagnosis. However, the former authors reported an elevated risk for suicide attempts only in ADHD males with more severe symptoms. In contrast, Chronis-Tuscano et al. (23) evaluated a greater risk for suicide attempts for girls after the first year of follow-up with Cox modeling, when the participants were only 5–7 years old. This limited finding raises the question on the severity of symptoms and the number of suicidal thoughts that may vary at different rates during the course of growing up. In our results, the importance of monitoring suicidal thought and behavior emerges, especially in individuals with ADHD in both genders. However, there are methodological differences between these studies, especially in terms of the sex ratio and the methodology of ADHD diagnosis vs. symptoms monitoring.

Focusing on the persistence of ADHD, two studies were included in our review (32, 34); however, the data of both studies were derived from the same female population (37). Interesting findings suggest that patients with persistent ADHD symptoms showed a greater risk for suicide thought and behavior. However, as these studies included only female participants and did not include ADHD participants only with hyperactivity symptoms, further studies are needed in the future.

In the case of ADHD subtypes, Chronis-Tuscano et al. (23) found a greater risk for suicide in the combined ADHD group only. Surprisingly, the HI and IT subtypes showed no elevated risk for future suicide thought and behavior. Consistent with this finding, Swanson et al. (34) reported an elevated suicide risk only in ADHD-C type, although the sample did not include ADHD-HI participants and boys.

These limited findings raise the possibility that children with ADHD may form an inhomogeneous group as a possible suicide risk factor. Our findings suggest that ADHD comorbidities, especially MD, combined type of ADHD, and persistence of the symptoms play a significant role in future suicide thought and behavior.

Long-term data about the effect of ADHD treatment on suicidality in childhood or adolescence are limited. Only one study (63) examined the effect of ADHD drug treatment and medication (methylphenidate and atomoxetine) on suicidality and found that it did not increase the risk of a suicide attempt. Our limited results also highlighted the importance of early detection and adequate treatment of ADHD cases.

Based on our review, we have the following methodological suggestions for future studies: (1) monitoring the ADHD symptom persistence and severity, (2) using more sophisticated suicidal thought and behavior (including thoughts, intentions, plans, and attempts) measurement tools, (3) monitoring the treatment of ADHD at a longitudinal setting, and (4) identifying the comorbidities, especially of MD, in participants.

## Limitations

Among the selected studies, only one published data about the treatment of ADHD highlighted the importance of ADHD treatment which could even decrease the symptoms of depression and the number of harmful outcomes (64), including suicide thought and behavior. Our results are limited for us to be able to evaluate the effect of ADHD treatment on suicidality.

Further limitations are derived from the method of the study selection. Firstly, among the search terms that we used, the ADHD terms to find the relevant articles with participants who met the ADHD diagnosis did not minimize the symptoms-only related articles. Secondly, we performed the database search with terms of suicidal behavior. We did not add the terms “self-harm” or “self-injury” to focus on suicidal topics and minimize the articles that investigated non-suicidal self-harm or self-injury. These limitations are the cause that we may be missing some relevant studies on this topic.

## Conclusion

In conclusion, our results, based on follow-up studies, underline the growing evidence of higher later suicidal risk of children and adolescents with ADHD. In the future, there is an additional need to clarify the role of treatment, comorbidities, subtypes, and persistence of ADHD in suicide thought and behavior. We would like to highlight the importance of the early diagnosis and treatment of ADHD, and during treatment, we need to pay special attention to comorbid conditions, especially anxiety and depression symptoms in boys.

## Data Availability Statement

All datasets generated for this study are included in the article/supplementary material.

## Author Contributions

PG: article selection process, presentation of the results, and discussion. JB: supervising the selection process and the presentation of the results and discussion. All authors contributed to the article and approved the submitted version.

## Conflict of Interest

The authors declare that the research was conducted in the absence of any commercial or financial relationships that could be construed as a potential conflict of interest.
